# Carrier-free nanomodulators reprogramming acetate metabolism for cancer photo-immunotherapy via c-Myc inhibition-induced closed-loop regulatory circuit

**DOI:** 10.1016/j.mtbio.2026.103660

**Published:** 2026-09-10

**Authors:** Jianwei Zhu, Danming Dong, Jiayue Xu, Xiaopeng Tang, Yuan Gao, Jinbin Zhu, Yini Zhuang, Pei Zhang, Zhuang Lv, Xiaoyuan Chen, Jianhua Zou, Huaxia Shi

**Affiliations:** a Department of Biochemistry, School of Life Sciences, Nanjing Normal University, Nanjing, Jiangsu, 210046, China; b Jiangsu Key Laboratory of New Drug Research and Clinical Pharmacy, Xuzhou Medical University, Xuzhou, Jiangsu, 221004, China; c Center for Nuclear Medicine and Molecular Imaging (CNMMI), Shandong Cancer Hospital and Institute, Jinan, China; d Department of Biochemistry, School of Medicine, Southeast University, Nanjing, Jiangsu, 210009, China

**Keywords:** Carrier-free nanoparticles, Acetate metabolism, c-Myc inhibition, Closed-loop regulatory circuit, Photo-immunotherapy

## Abstract

Non-small cell lung cancer (NSCLC) remains difficult to cure owing to therapy resistance and immune evasion that limits durable therapeutic responses. Here, we report a carrier-free nanoregulator (CE NPs) formed by co-assembling chlorin e6 (Ce6) with a covalent c-Myc inhibitor (EN4), enabling photo-immunotherapy that couples photodynamic therapy (PDT)-induced immunogenic cell death (ICD) with acetate-metabolism-driven immune reprogramming. Upon 660-nm light irradiation, CE NPs efficiently generate reactive oxygen species to trigger apoptosis and subsequent ICD. In parallel, EN4-mediated c-Myc suppression coordinately downregulates MCT1, Cyclin D1, LDHA, and PD-L1, thereby interrupting the MCT1/acetate/acetyl-CoA/c-Myc self-amplifying “closed-loop” circuit, restraining tumor proliferation and glycolysis, and alleviating PD-L1-associated immune escape. Consequently, CE NPs, along with 660-nm light illumination, achieve robust tumor growth inhibition without obvious systemic toxicity under the tested conditions, promotes systemic immune activation, suppresses distant tumors in a bilateral model, and establishes durable immune memory that resists tumor rechallenge. Collectively, this work establishes a “closed-loop regulatory circuit” strategy by integrating PDT-induced ICD with acetate-metabolism reprogramming to overcome immune evasion and improve NSCLC photo-immunotherapy.

## Graphical abstract

**Figure:**
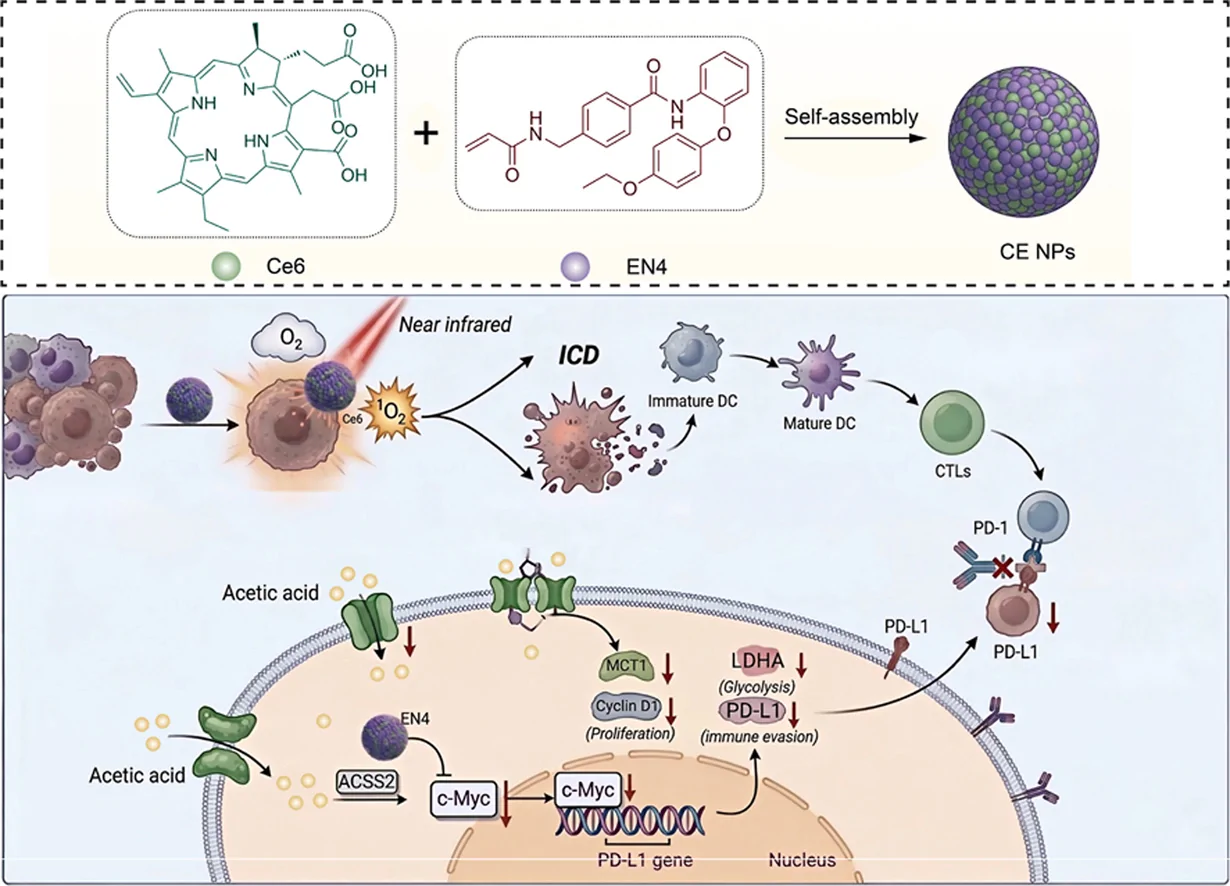

## 1 Introduction

Non-small cell lung cancer (NSCLC) remains one of the most lethal malignancies worldwide, largely because of various challenges towards treatments [[1], [2], [3], [4]]. For example, surgery for NSCLC suffers from recurrence and metastasis [5,6]. Chemotherapy or radiotherapy for NSCLC is often limited by resistance and severe side effect [7,8]. Immunotherapy shows great potential in cancer treatment by harnessing the patient's own immune system to eliminate cancers [9,10]. More importantly, immunotherapy also inhibits tumor metastasis and produce durable, long-lasting responses [11,12]. However, NSCLS is usually resistant to immunotherapy because it maintains an immune-cold microenvironment with weak antigen presentation and limited T cell infiltration [[13], [14], [15]]. In addition, various studies have confirmed dual-modality or multi-modality treatment is obviously superior to single treatment modality [16].

In recent years, photodynamic therapy (PDT) appears a new and non-invasive cancer treatment, which activates photosensitizers (PSs) with light illumination to generate reactive oxygen species (ROS) and directly kill tumors locally [17]. Compared to conventional therapies, PDT offers spatiotemporal control and typically causes fewer systemic side effects because it is only triggered where and when light is applied [18]. More importantly, PDT can also induce immunogenic cell death (ICD), releasing tumor antigens and danger signals to help prime antitumor immunity, making it an attractive strategy to both ablate tumors and boost antitumor immune responses [19]. Despite that, the overall immune response is often suppressed due to cold immune microenvironment, causing immune checkpoint blockade and limited T-cell infiltration [20,21].

Recently, metabolic rewiring has been increasingly recognized as an important driver of tumor immune suppression [22]. Extensive studies have demonstrated the significant influence of glucose and lactate metabolisms on the tumor immune microenvironment [23,24]. Interestingly, acetate metabolism has emerged as another important metabolic pathway involved in tumor progression and immune regulation [[25], [26], [27], [28]]. In particular, Wang et al. reported that lung cancer cells uptake acetate via the transporter MCT1 to maintain intracellular acetyl-CoA levels [29]. This acetate flux triggered the acetylation and stabilization of c-Myc, a key factor for gene expression regulation [29]. The increasing level of c-Myc in turn boosting MCT1 transcription to form an acetate metabolism based “closed-loop” regulatory circuit and particularly upregulates PD-L1 expression, ultimately suppressing CD8^+^ T cell activity and driving immune evasion in NSCLC [29].

In the meanwhile, carrier-free nanomedicines provide a simple platform for integrating multiple therapeutic functions without additional carriers [30,31]. Previous studies have combined carrier-free PDT with regulating lactic-acid-associated metabolism or modulating PD-L1-associated immune response [32,33]. However, therapeutic targeting of the upstream c-Myc-centered acetate metabolic feedback circuit, together with PDT-induced ICD, remains largely unexplored.

**Scheme 1:**
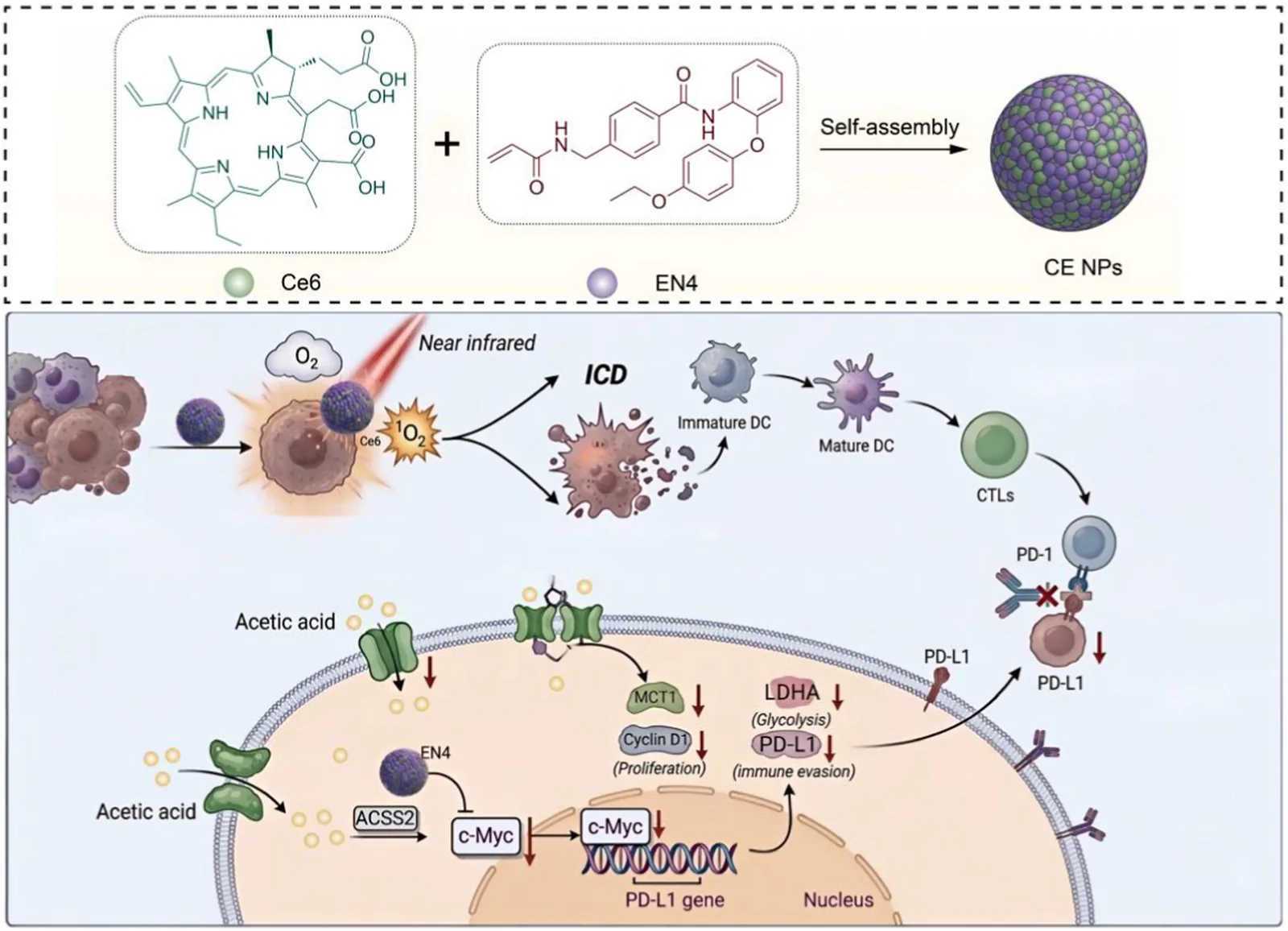
Schematic illustration of the design rationale and proposed working mechanism of CE NPs for cancer photo-immunotherapy.

Herein, we developed a carrier-free nanoplatform (CE NPs) that integrated PDT-induced ICD and acetate metabolism-based PD-L1 downregulation for NSCLC photo-immunotherapy. CE NPs were co-assembled by the photosensitizer chlorin e6 (Ce6) and EN4, a c-Myc inhibitor without any carriers (Scheme 1). Upon 660-nm light illumination, CE NPs generated ROS to induce cellular apoptosis and further cause ICD to promote tumor antigen presentation and dendritic cell maturation to boost immune responses. In the meanwhile, CE NPs inhibited c-Myc expression, further downregulating levels of MCT1, Cyclin D1, LDHA, and PD-L1 simultaneously. Decreasing Cyclin D1 expression resulted in NSCLC proliferation inhibition while reducing MCT1 prevented acetate influx into NSCLC and further disrupted the acetate metabolism based “closed-loop” regulatory circuit (Scheme 1). More importantly, PD-L1 downregulation induced by c-Myc inhibition facilitated effective CD8^+^ T-cell responses and avoid immune evasion. In vivo results showed CE NPs not only achieved robust tumor growth inhibition with light illumination, but also remodeled the tumor immune landscape, enabling systemic immune activation, distal tumor growth control, and durable antitumor immune memory. In summary, our study provides a promising avenue to reprogram acetate metabolism to overcome the immune evasion and improve the NSCLC photo-immunotherapy.

## 2 Results and discussion

**Fig. 1:**
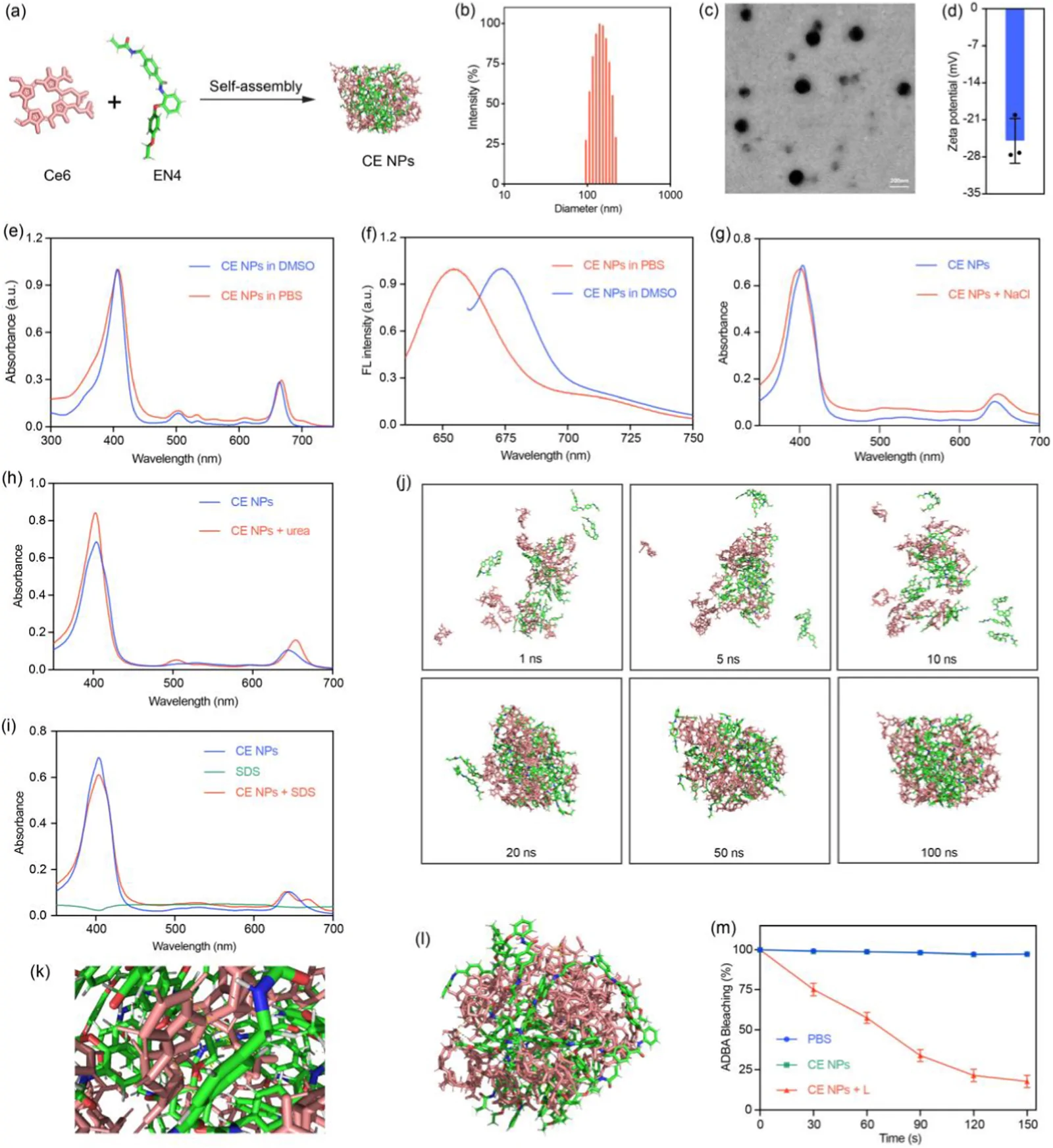
Preparation, characterization, and mechanism exploration of CE NPs. (a) Schematic illustration of Ce6 and EN4 assembled into CE NPs via intermolecular interactions. (b) DLS size distribution of CE NPs. (c) Representative transmission electron microscopy image of CE NPs. Scale bar: 200 nm. (d) Zeta potential of CE NPs, n = 3. (e) The UV-vis absorbance spectrum and fluorescence emission spectrum (f) of CE NPs in PBS and DMSO. UV-vis absorbance spectrum of CE NPs in the presence or absence of NaCl (g), urea (h), and SDS (i). (j) MD simulations of Ce6 and EN4 to self-assemble into CE NPs over 100 ns time scale. Intermolecular interaction patterns like hydrogen bonding (l) and π-π stacking (k) in CE NPs system. (m) Singlet oxygen generation (^1^O_2_) of CE NPs as under 660-nm light illumination (power density: 0.5 W/cm^2^) using ABDA as ^1^O_2_ probe. PBS only and CE NPs without light illumination were served as control, n = 3. Data are presented as mean ± SD.

Ce6 and EN4 were co-assembled into carrier-free nanoparticles (CE NPs) through intermolecular interactions owning to large planar aromatic porphyrin and three carboxy groups of Ce6 (Fig. 1a) [[30], [31], [32]]. Considering the fact that feed ratio is a key factor for carrier-free nanoassembly, we next examined the influence of Ce6: EN4 ratio on nanoparticle formation [32]. Dynamic light scattering (DLS) confirmed the hydrodynamic size of nanoparticles was around 150 nm when the Ce6: EN4 molar ratio was 1: 1 (Fig. 1b). When feeding ratio was adjusted to 1:2 or 1:3, the as-obtained nanoparticles became larger and ununiform (Fig. S1). Further transmission electron microscopy (TEM) showed the morphology of CE NPs with the feeding ratio of 1:1, supporting successful formation of nanoparticles (Fig. 1c). Thus, CE NPs with feeding ratio of 1:1 for Ce6: EN4 was selected for the subsequent studies. In addition, the as-prepared nanoparticles exhibited the negative surface charge with zeta potential around −20 mV (Fig. 1d). Stability of nanoparticles is a significant factor for their usage in cancer theranostics. We then evaluated the stability of CE NPs by monitoring their DLS size and PDI. CE NPs remained largely stable for particle size and PDI in both H_2_O and PBS over 7 days (Fig. S2), demonstrating the stable property of CE NPs under both physiological or non-physiological condition.

Next, UV-vis spectra showed the characteristic bands of CE NPs in PBS and DMSO, showing a slight shift around 660 nm (Fig. 1e). However, fluorescence emission of CE NPs was markedly blue-shifted in PBS compared to that in DMSO, mainly attributing the aggregation of Ce6 and EN4 in PBS (Fig. 1f). To probe how noncovalent forces the aggregation of CE NPs, we analyzed the UV-vis spectra of CE NPs treated with different electrolytes or denaturants. The UV-vis spectra of CE NPs in the presence of NaCl did not change significantly, indicating the negligible electrostatic interaction in CE NPs (Fig. 1g). Instead, in a high-concentration urea solution, the UV-vis spectrum of CE NPs changed obviously, revealing that the hydrogen-bond interaction was the acting force in CE NPs formation (Fig. 1h). Moreover, the absorption at around 660 nm changed with the addition of SDS, further confirming that hydrophobic interactions also promoted the self-assembly of CE NPs (Fig. 1i). Together, based on these findings, Ce6 and EN4 were assembled into CE NPs through hydrophobic interaction, π-π interaction, and hydrogen bond interaction.

To elucidate the assembly process at the molecular level, molecular dynamics (MD) simulations were conducted. Time-resolved snapshots showed that Ce6 and EN4 rapidly reorganized from a dispersed state into compact aggregates and evolved into a stable nanoparticle-like assembly over the 100 ns trajectory (Fig. 1j). Structural inspection of the equilibrated ensemble revealed abundant intermolecular contacts, including hydrogen-bonding networks (Fig. 1k) and π-π stacking between aromatic moieties (Fig. 1l). Thus, MD analyses demonstrate the self-assembly of Ce6 and EN4 to form nanoparticles are driven mainly by hydrogen bonds and π-π stacking in aqueous environments.

Subsequently, we continued to explore whether CE NPs retained singlet oxygen generation ability after formation of nanoparticles. To do so, ABDA (9, 10-anthracenediyl-bis(methylene)dimalonic acid) was employed as a probe of singlet oxygen [34,35]. As Ce6 is a well-established photosensitizer with efficient ^1^O_2_-generating capability, the ABDA assay was used here to verify whether this photodynamic activity was retained after its co-assembly with EN4 into CE NPs. More ABDA bleaching quantified by fluorescence intensity means more singlet oxygen generated. Upon light illumination, fluorescence intensity of ABDA in the presence of CE NPs was gradually decreased with time prolonging while that for PBS or CE NPs alone remained unchanged (Fig. 1m). This result confirmed CE NPs possess the ability to generate singlet oxygen with light illumination for further photodynamic cancer treatment.

**Fig. 2:**
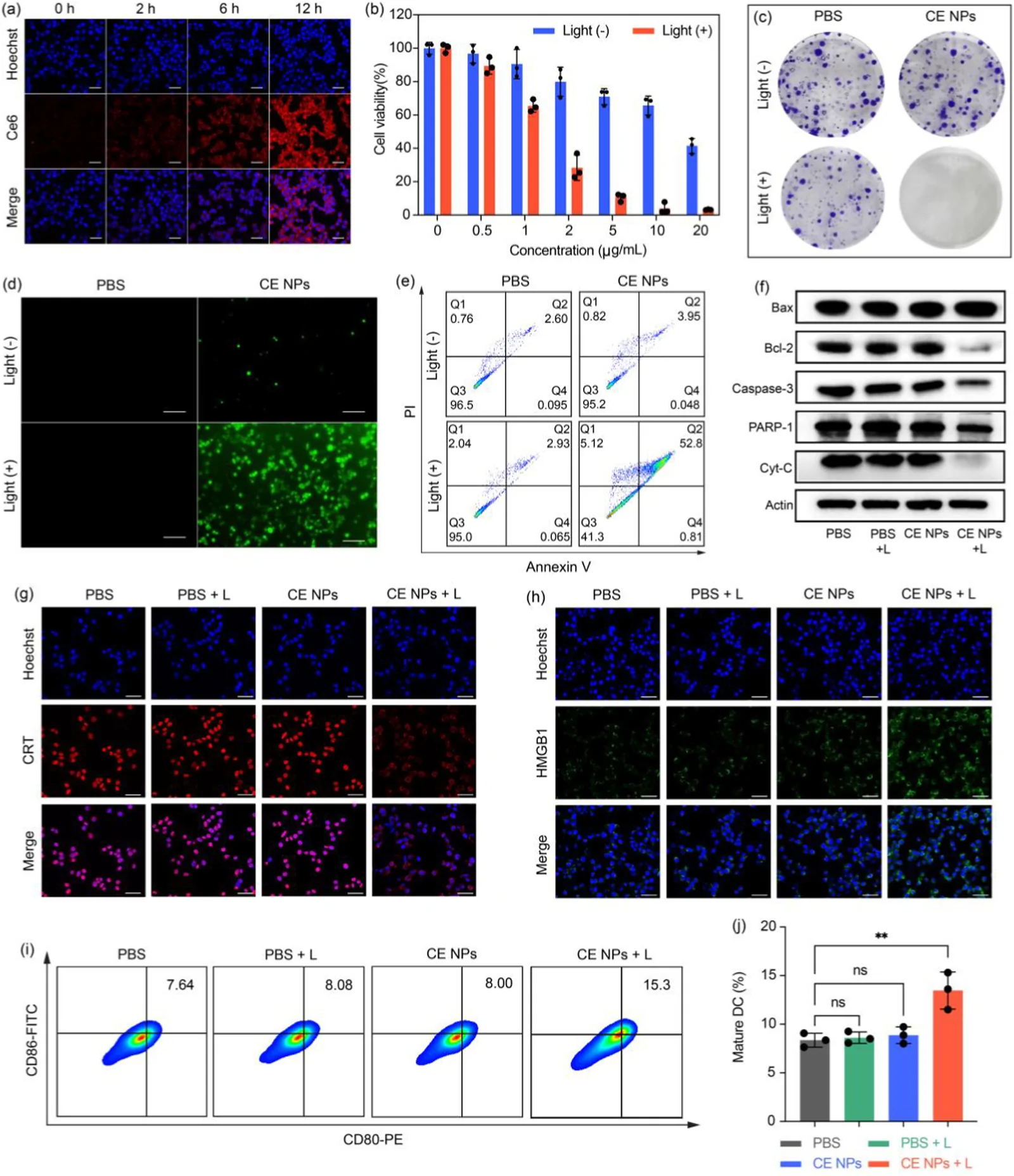
In vitro antitumor efficiency and mechanism of CE NPs under light illumination. (a) Confocal laser scanning microscopy (CLSM) images of intracellular uptake of CE NPs in LLC cells. Scale bar: 10 μm. (b) Cell viability of LLC cells treated with CE NPs at different concentrations with or without light illumination. (c) Colony formation images of LLC cells with different treatments, including PBS, PBS + L, CE NPs, and CE NPs + L. (d) Fluorescence images of intracellular ROS generation in LLC cells with different treatment by using DCFH-DA as probe. Scale bar = 100 μm. (e) The population of apoptosis cells treated with PBS and CE NPs with or without light illumination via Annexin V-FITC/PI double-staining assay using FCM analysis. (f) Western blotting analysis for Bax, Bcl-2, Caspase-3, PARP-1, and Cytochrome C protein expressions in LLC cells with different treatments. β-actin serves as control. CLSM images of CRT exposure (g) and HMGB1 release (h) in LLC cells after different treatment. FCM analysis (i) and quantitative analysis (j) of DCs after co-incubating with LLCs with different treatment. Quantitative data are presented as mean ± SD from three independent experiments (n = 3). Statistical analysis of cell viability in (b) was performed using two-way ANOVA. For comparisons among the four treatment groups in the other quantitative analyses, one-way ANOVA was used. Statistical significance was analyzed as indicated: ns, not significant; **P < 0.01.

Previous studies have demonstrated that nanoparticle-based pro-drugs enhance their internalization efficiency in cells compared with free Ce6. To verify whether CE NPs could be internalized by LLC cells, confocal laser scanning microscopy (CLSM) was employed to evaluate the cellular uptake behavior of CE NPs. The red fluorescence emission of CE NPs gradually increased with time prolonging and reached the maximum intensity at 12h, indicating intracellular internalization of CE NPs was time-dependent (Fig. 2a). Similar tendence was observed via flow cytometry (FCM), further confirming that CE NPs can be effectively up-taken by LLC cells (Fig. S3). Ce6 is reported as an effective photosensitizer to trigger PDT for cancer treatment. To evaluate the in vitro antitumor activity of CE NPs, cell viability was measured and analyzed using a Cell Counting Kit 8 (CCK8) by incubating LLC cells with different concentrations of CE NPs with or without light illumination. Under light illumination, cell viability decreased gradually with the increasing concentration and dramatically dropped to ∼25% at the concentration of 2 μg/mL, indicating the excellent antitumor activity of CE NPs with light illumination (Fig. 2b). Interestingly, cell viability also decreased with the concentration increased even without light illumination (Fig. 2b), revealing possible antitumor ability of EN4 inside CE NPs. Further colony formation assay confirmed that CE NPs perform best to inhibit the proliferation of LLC cells with light illumination (Fig. 2c), consistent with the findings from CCK8 assays in Fig. 1b. Overall, these results suggest CE NPs possess excellent antitumor activity with light illumination.

To explore how CE NPs inhibit the growth of LLC cells with light, intracellular ROS generation was studied by using 2′,7′-dichlorodihydrofluorescein diacetate (DCFH-DA) as a ROS probe [35]. Typically, non-fluorescent DCFH-DA reacts with intracellular ROS to generate 2′,7′-dichlorofluorescein (DCF). As expected, 660-nm light illumination increased the fluorescence obviously in LLC cells treated with CE NPs compared with the rest three groups (Fig. 2d and S4), confirming that CE NPs with light illumination elevate the ROS levels in LLC cells. This result is consistent with that obtained in PBS (Fig. 1l).

Previous studies have reported that increased ROS can induce apoptosis in cancer cells [35]. To investigate when CE NPs cause cellular apoptosis with light illumination, flow cytometry was applied via annexin V-FITC/propidium iodide (PI) double-staining. CE NPs + L induced the highest apoptosis rate of ∼53.57% while CE NPs only induced very low apoptosis rate closed to the other two control groups (Fig. 2e and S5). In addition, western blotting further showed treatment-associated changes in apoptosis-related proteins, including increased Bax and decreased Bcl-2, together with alterations in caspase-3, PARP-1, and cytochrome *c* levels (Fig. 2f and Fig. S6). Together with the flow cytometry (Fig. 2e and S4), these results support the induction of apoptosis by CE NPs under 660-nm light illumination.

Photodynamic cancer therapy may induce immunogenic cell death (ICD) effect and stimulate adaptive antitumor immunity by triggering the release of various DAMPs (e.g. CRT and HMGB1) from dying tumor cells [32,36]. Generally, CRT is released from endoplasmic reticulum (ER) to the cell membrane surface as the signal of “eat me” while HMGB1 moves from the nucleus as the signal of “find me” to promote phagocytosis and subsequently recruit CTLs [32,37,38]. To explore whether CE NPs + L induce ICD effect, CLSM was employed to monitor the expression levels of CRT and HMGB1 by an immunofluorescence approach. CE NPs + L treatment induced the enhanced CRT expression on the cell membrane surface compared to other three groups (Fig. 2g), indicating PDT induced by CE NPs + L significantly increased CRT expression and may further facilitate the phagocytosis of dead tumor cells and their debris via immature DCs and macrophages. Besides that, LLC cells in CE NPs + L group exhibited the weakest HMGB1 fluorescence emission, indicating CE NPs-induced PDT stimulated the migration of HMGB1 from the nucleus and its extracellular release (Fig. 2h). Overall, both CRT exposure and HMGB1 release revealed the induction of ICD effect by PDT of CE NPs, which plays an important role in promoting DC maturation in the lymph nodes.

To further determine whether CE NPs-induced ICD through PDT could boost antigen presentation, we continued to explore the change of dendritic cell (DC) maturation by co-incubating immature DCs with LLC cells subjected to different treatments, followed by flow-cytometric analysis of DC maturation markers (CD80 and CD86). DCs exposed to LLC cells from CE NPs + L group exhibited a significantly increase in the CD80^+^CD86^+^ population compared with the rest three groups, indicating efficient induction of DC maturation (Fig. 2i and j). Collectively, CE NPs + L induced PDT and ICD effect to further boost DC maturation for further antitumor immune activation.

**Fig. 3:**
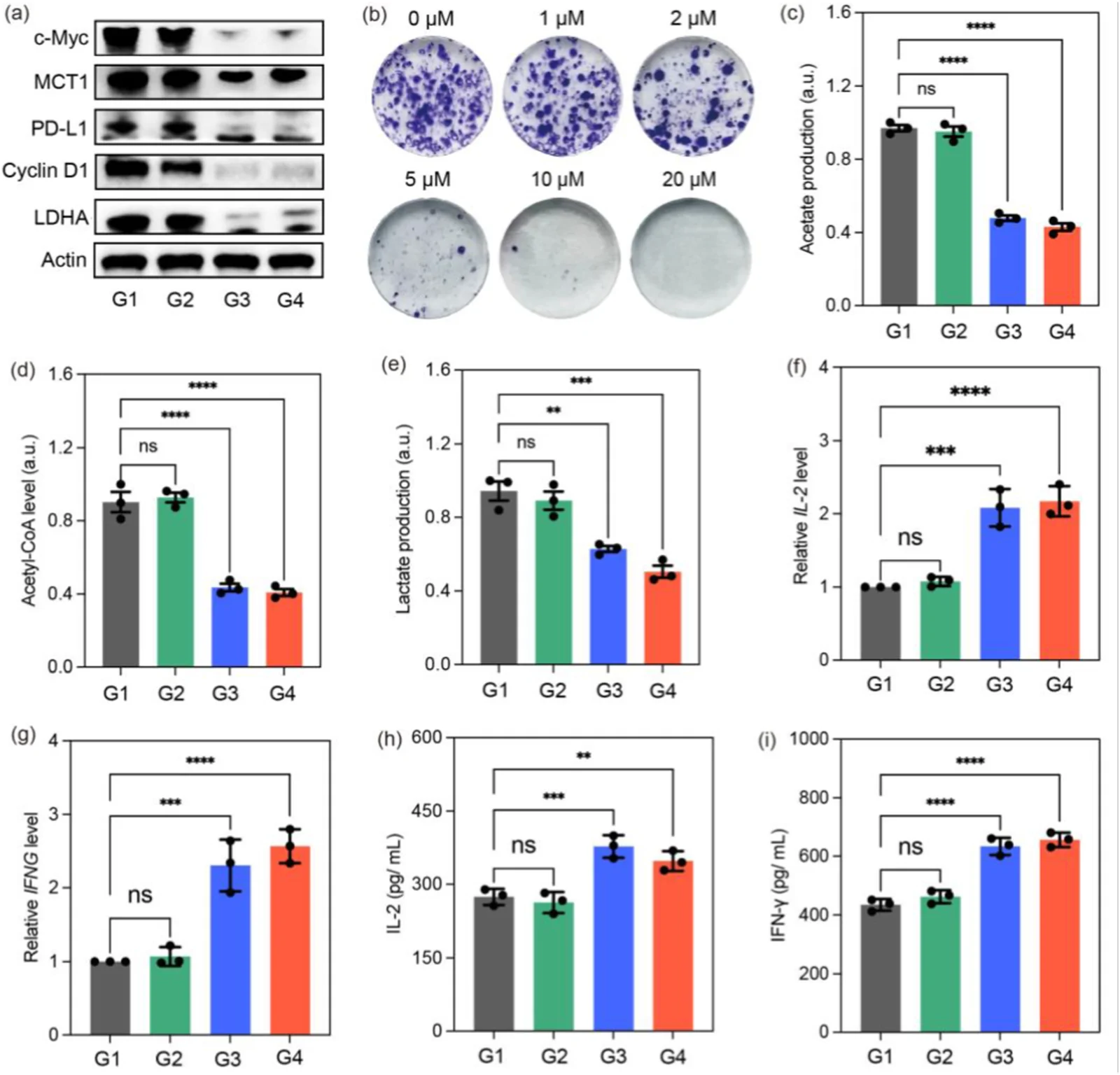
In vitro acetate-metabolism rewiring and immune reprogramming induced by CE NPs. (a) Protein expression was evaluated by western blotting in LLC cells with different treatment in low-glucose medium, including c-Myc, MCT1, PD-L1, Cyclin D1, and LDHA. β-actin serves as control. (b) Colony formation of LLC cells treated with increasing concentrations of CE NPs (0-20 μM) in low-glucose medium supplemented with a fixed concentration of acetate (1.0 mM). Acetate (c), acetyl-CoA (d) and lactate (e) levels in LLC cells with different treatment quantified by LC-MS. Relative IFNG (f) and IL-2 (g) expression level in LLC-OVA cells after different treatments. IL-2 (h) and IFN-γ (i) levels measured by ELISA in the co-culture system after the indicated treatments. G1: PBS, G2: PBS + L, G3: CE NPs, G4: CE NPs + L. Quantitative data are presented as mean ± SD from three independent experiments (n = 3) from (c) to (i). For comparisons among G1-G4 in the quantitative analyses, one-way ANOVA was used. Statistical significance was analyzed as indicated: ns, not significant; **P < 0.01, ***P < 0.001, ****P < 0.0001.

Recently, acetate is reported to regulate tumor metabolism and promote immune evasion via the MCT1-acetate-c-Myc self-boosting circuit, highlighting c-Myc as a potential therapeutic target for cancer treatment [29]. We wondered whether CE NPs were able to reprogram acetate metabolism and enhance the immunotherapeutic efficacy through direct inhibition of c-Myc. Western blotting results revealed CE NPs induced a coordinated downregulation of c-Myc and its downstream effector, MCT1 (Fig. 3a and S7), suggesting that EN4 released from CE NPs effectively suppressed the c-Myc-driven transcriptional program.

MCT1 is required for acetate uptake, which further sustains intracellular acetyl-CoA and promotes c-Myc stability in NSCLC [29]. We asked that downregulation of MCT1 may disrupt acetate influx and subsequently collapse the above closed-loop regulatory circuit. Wang et al. demonstrated NSCLC relied more on acetate supplementation for growth and proliferation under low-glucose condition [29]. Based on that, LLC cells were cultured in low-glucose medium supplemented with a fixed concentration of acetate (1.0 mM), and then treated with increasing concentrations of CE NPs. CE NPs suppressed colony formation in a concentration-dependent manner even in the presence of acetate (Fig. 3b), indicating that CE NP-mediated c-Myc inhibition can effectively counteract acetate-supported tumor cell growth.

Consistent with MCT1 downregulation, LC-MS analysis revealed that CE NPs markedly decreased intracellular acetate and acetyl-CoA levels compare with control groups (Fig. 3c and d). In parallel, CE NPs also reduced lactate levels (Fig. 3e), consistent with the reported role of the MCT1/c-Myc axis in regulating LDHA expression and lactate metabolism in NSCLC cells [29]. These results support that CE NPs restrain acetate flux via downregulating MCT1 and subsequent acetate metabolism by targeting the c-Myc-centered closed-loop circuitry.

Besides that, Cyclin D1 and LDHA are also two downstream effectors affected by c-Myc regulation, which are responsible for tumor proliferation and glycolysis, respectively [29]. Indeed, LLC cells treated with CE NPs exhibited the decreased Cyclin D1 and LDHA protein levels. These coordinated proliferative and metabolic constraints offered a mechanistic sight on the light-independent growth inhibition (“dark toxicity”) of CE NPs in Fig. 2b, revealing that CE NPs-mediated c-Myc suppression restricted both carbon utilization and proliferation capacity in LLC cells.

More importantly, c-Myc is a key transcription factor to regulate the gene expression of CD274 (encoding PD-L1) for tumor immune evasion [29,39]. Our western blotting results confirmed that CE NPs-mediated suppression of c-Myc decreased protein levels of PD-L1 compared with control group in LLC cells (Fig. 3b). However, how CE NPs regulate T cell activation still remains elusive. To do so, ovalbumin (OVA)-expressing mouse LLC (LLC-OVA) cells were treated with acetate, followed with CE NPs or PBS under different conditions. Next, these treated LLC-OVA cells were co-cultured with CD8^+^ T cells derived from OT-1 mouse. Interestingly, CE NPs-treated cells promoted the mRNA (Fig. 3f and g) and protein expression (Fig. 3h and i) of interleukin (IL)-2 and interferon (IFN)- γ (encoded by IFNG) in co-cultured CD8^+^ T cells compared to those in CE NPs-untreated cells, revealing that CE NPs-mediated PD-L1 downregulation increased CD8^+^ T cell activation in vitro. Overall, these results demonstrate that CE NPs-induced c-Myc inhibition reprograms acetate metabolism, inhibits tumor proliferation, and decreases PD-L1 to activate CD8^+^ T cell.

**Fig. 4:**
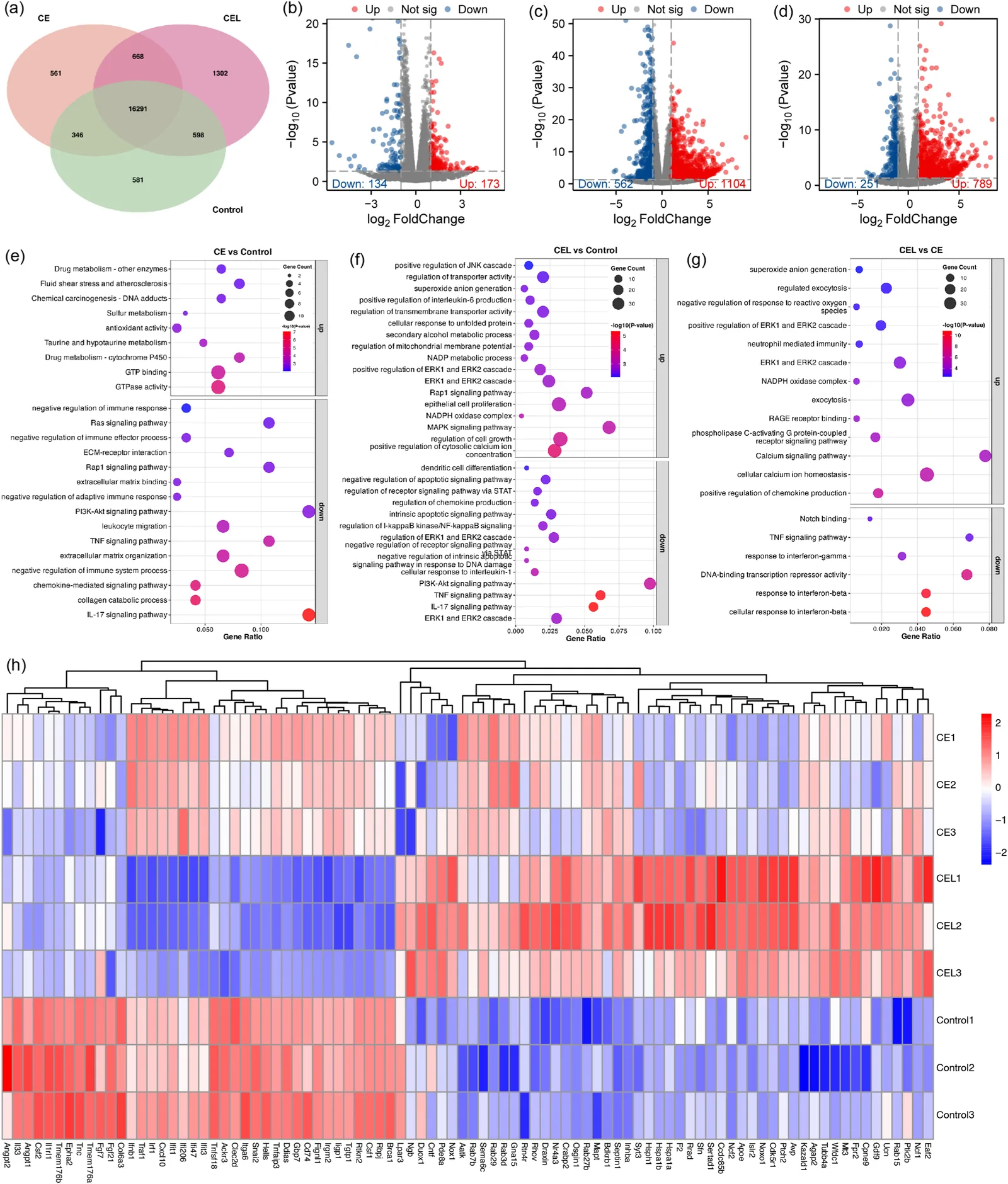
Transcriptomic RNA sequencing in LLC cells treated with CE NPs (CE) and CE NPs + L (CEL) (n = 3). (a) Venn diagram illustrating the number of genes transcribed in each group (CE, CEL, and Control). Volcano plots representing the differentially expressed genes (DEGs) between each group: CE vs Control (b), CEL vs Control (c), and CE vs CEL (d). Kyoto Encyclopedia of Genes and Genomes (KEGG) enrichment analysis among: CE vs Control (e), CEL vs Control (f), and CE vs CEL (g). (h) Differential gene clustering heat map of different samples.

To further investigate the anti-cancer mechanism of CE NPs, the transcriptomic RNA sequencing was performed on LLC cells treated with PBS (Control), CE NPs (CE), and CE NPs + L (CEL), respectively. A Venn diagram exhibited the overlap of expressed genes among the three groups, indicating preserved basal transcriptional activity (Fig. 4a). In specific, CEL displayed a larger set of uniquely transcribed genes compared with CE, suggesting a main light-illumination mediated transcriptional reprogramming (Fig. 4a). Consistently, differential expression analysis showed that CE elicited a relatively modest transcriptional shift versus Control (173 upregulated and 134 downregulated genes), whereas CEL induced a markedly broader response (1104 upregulated and 562 downregulated genes) (Fig. 4b and c). Direct comparison between CE and CEL further confirmed that light illumination introduced a distinct gene expression signature (789 upregulated and 251 downregulated genes), separating the photoactivated response from CE-alone effects (Fig. 4d).

Pathway enrichment analyses corroborated these dual-mode mechanisms. In the CE vs Control comparison, KEGG analysis highlighted enrichment of metabolic adaptation and signaling pathways associated with nutrient stress and growth regulation, including multiple survival/proliferation-linked signaling modules (e.g., Ras/Rap1/PI3K-Akt) together with inflammatory/immune-regulatory networks such as TNF and IL-17 signaling, chemokine-related processes, and extracellular matrix-associated pathways (Fig. 4e). This transcriptional pattern is consistent with CE-induced metabolic rewiring under low-glucose conditions, aligning with the observed suppression of the acetate/c-Myc metabolic axis and its downstream proliferation/immune-evasion outputs (e.g., PD-L1 associated regulation) in subsequent mechanistic assays.

In contrast, CEL vs Control enrichment profiles were dominated by oxidative stress- and cell death-associated programs. Notably, pathways linked to ROS production and redox stress (e.g., superoxide anion generation and NADPH oxidase-related modules), stress-activated kinase cascades (MAPK and ERK/JNK-associated signaling), and apoptosis-related processes were prominently enriched (Fig. 4f). These transcriptomic features provide system-level support for the light-triggered ROS burst and apoptosis observed experimentally. Importantly, CEL also exhibited strong activation of immune-instructive signaling, including TNF/IL-17 associated inflammation and interferon-responsive programs, which are frequently associated with enhanced antigen presentation and immunogenic cell death (ICD)-related immune priming.

When isolating the light-dependent contribution (CE vs CEL), KEGG enrichment further reinforced the emergence of immune-activating and ICD effect upon photoactivation. Compared with CE alone, CEL showed enrichment of interferon-associated responses (including interferon-γ/β response terms), positive regulation of chemokine production, and inflammatory signaling (e.g., TNF), alongside membrane/secretory processes potentially linked to damage-associated signal (Fig. 4g). Finally, unsupervised clustering and heatmap analysis demonstrated clear segregation of Control, CE, and CEL samples with high within-group consistency (Fig. 4h), confirming robust transcriptional state transitions.

Collectively, these transcriptomic results suggest that the two functional modules of CE NPs contribute to tumor suppression through complementary mechanisms. EN4-mediated c-Myc inhibition primarily regulates tumor-intrinsic metabolic and immune-evasion programs, including acetate metabolism-associated pathways and PD-L1 expression. In contrast, Ce6-mediated photodynamic activation induces oxidative stress-associated cell death and immune-related signaling, providing an immunogenic stimulus for subsequent immune activation. Therefore, the metabolic regulation and PDT-induced ICD modules are not simply additive components but function in a complementary manner, with metabolic reprogramming reducing tumor immune escape while PDT providing immune priming signals.

**Fig. 5:**
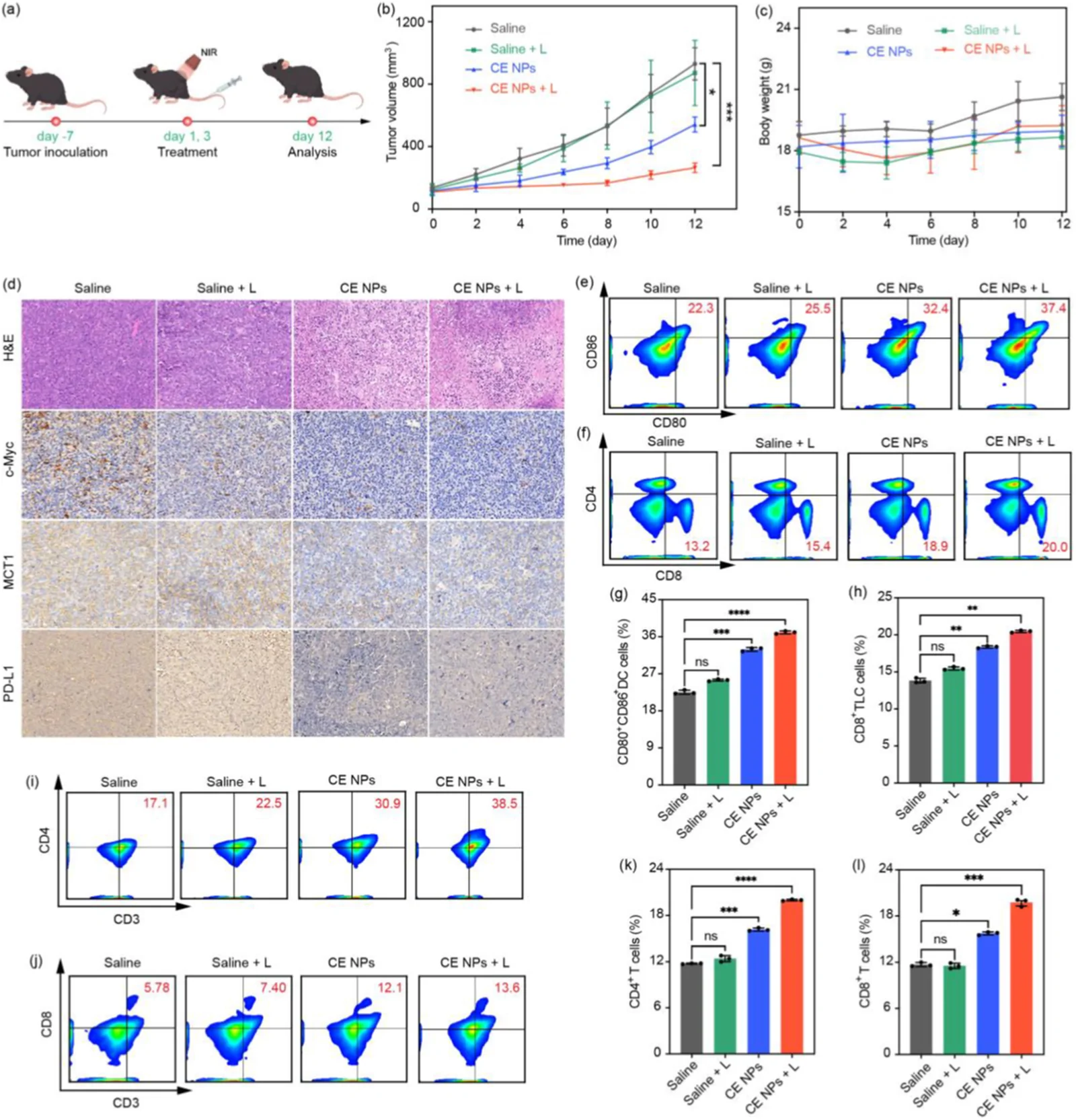
*In vivo* antitumor efficacy and immune activation induced by CE NPs under 660-nm light illumination. (a) Schematic illustration of the LLC tumor-bearing mouse model and treatment schedule. Tumor growth curves (b) and body weight (c) of mice treated with Saline, Saline + L, CE NPs, and CE NPs + L (n = 5). (d) H&E staining and immunohistochemical (IHC) staining of c-Myc, MCT1, and PD-L1 in tumor tissues harvested from different groups at day 12. (e) Representative flow cytometry plots showing the frequency of mature dendritic cells (CD80^+^CD86^+^) in spleen. (f) Representative flow cytometry plots showing tumor-infiltrating CD8^+^ T in spleen. (g) Quantification of mature DCs (CD80^+^ CD86^+^) corresponding to (e). (h) Quantification of CD8^+^ T cells corresponding to (f). Representative flow cytometry plots (i) and quantification (k) of CD4^+^ T cells in tumors (n = 3). Representative flow cytometry plots (j) and quantification (l) of CD8^+^ T cells in tumors (n = 3). Light parameters: 660 nm, 0.5 W/cm^2^, 5 min, 150 J/cm^2^. Quantitative data are presented as mean ± SD. Tumor-growth and body-weight curves in (b) and (c) were analyzed using two-way repeated-measures ANOVA. For single-time-point comparisons among the four treatment groups, one-way ANOVA was used. Statistical significance was analyzed as indicated. ns, not significant; *P < 0.05, **P < 0.01, ***P < 0.001, ****P < 0.0001.

We next evaluated the therapeutic performance of CE NPs in an LLC tumor-bearing mouse model following the treatment schedule illustrated in Fig. 5a. To determine the optimal time for light illumination, in vivo fluorescence imaging was employed to assess the accumulation of CE NPs in LLC tumor-bearing mice after intravenous injection. Following injection, the fluorescence intensity exhibited a gradual increase at the tumor sites, reaching its peak at 12 h post-injection and then tended to decrease with time prolonging (Fig. S8). Thus, 12-h post-injection was chosen for the subsequent experiments in vivo.

Tumor growth was slightly inhibited by CE NPs alone compared to Saline or Saline + L (Fig. 5b). Notably, the combination treatment (CE NPs + L) achieved the most effective tumor suppression with the smallest tumor volume at the endpoint (Fig. 5b), revealing the robust in vivo antitumor efficacy of CE NPs with 660-nm light illumination. To preliminarily evaluate systemic tolerability during this treatment period, body weight was also monitored. No significant body-weight loss was observed among each group over 12 days, indicating minimal systemic toxicity within treatment (Fig. 5c). In addition, serum biochemical markers were also investigated at day 12. Creatinine (CRE) and blood urea nitrogen (BUN) (two indicators of kidney function) showed no significant differences while aspartate aminotransferase (AST) and alanine aminotransferase (ALT) (two liver injury-associated enzymes) remain similar levels among all groups (Fig. S9). Taken together, these results confirm that CE NPs along with 660-nm light illumination exhibited superior therapeutic outcomes with negligible systemic toxicity under the tested conditions in vivo.

Histopathological assessment further supported the therapeutic benefit. H&E staining revealed substantially increased tissue damage or necrotic features in tumors treated with CE NPs compared with control groups (Fig. 5d) while the rest organs and tissues remain undamaged across all the groups (Fig. S10). In addition, immunohistochemical analyses demonstrated that CE NPs-based treatment attenuated the expression of representative proteins concerning acetate related metabolism in tumor tissues, including MCT1 and c-Myc (Fig. 5d). To further verify whether CE NPs could regulate acetate-associated metabolism in vivo, we additionally quantified acetate and acetyl-CoA levels in harvested LLC tumor tissues. Compared with saline-treated groups, CE NPs treatment significantly reduced intratumoral acetate and acetyl-CoA levels, while light irradiation alone showed minimal influence (Fig. S11 and S12). These results provide direct evidence that CE NPs can modulate acetate metabolism within tumor tissues.

More importantly, PD-L1 expression was also decreased after CE NPs treatment with or without light illumination compared to saline or saline + L group, revealing its potential in tumor immunotherapy (Fig. 5d). Besides that, consistent with in vitro ICD findings, tumor IHC further showed enhanced CRT staining and decreased HMGB1 retention in CE NPs + L group compared to other groups (Fig. S13), supporting that CE NPs-based phototherapy induces ICD in vivo and thereby facilitates downstream immune response.

Considering this, we next explored whether CE NPs + L could affect systemic and local immune activation in vivo. Flow cytometry analysis of splenic immune populations showed an increased frequency of mature dendritic cells (CD80^+^CD86^+^) following CE NPs treatment, which was further boosted with light illumination (Fig. 5e and g), indicating the strengthened antigen-presenting capacity at the systemic level. In the meanwhile, splenic CD8^+^ T cells were elevated in CE NPs group and reached the highest level in CE NPs + L group (Fig. 5f and h), supporting enhanced systemic cytotoxic T-cell priming and expansion induced by CE NPs, especially with light illumination.

To determine whether systemic immune activation was accompanied by reinforced tumor-local immune surveillance, tumor-associated T-cell populations were further analyzed by flow cytometry. Representative gating plots and quantification demonstrated that CE NPs treatment increased intratumoral CD4^+^ T cells and CD8^+^ T cells, and this effect was significantly amplified in CE NPs + L group (Fig. 5i and k). In addition, systemic effector cytokine levels in serum were also investigated by ELISA analysis. IFN-γ and TNF-α levels reached the highest levels in CE NPs + L group while those increased modestly in CE NPs groups compared with control group (Fig. S14). This result suggests that CE NPs-mediated metabolic reprogramming facilitates immune cytokine production, especially with 660-nm light immunination. Together, these results demonstrate that CE NPs + L exerts potent tumor growth inhibition by promoting systemic DC maturation, CD8^+^ T-cell expansion, and enhancing intratumoral T-cell infiltration, thereby supporting the therapeutic rationale of CE NPs as a photo-immunotherapeutic nanoplatform.

**Fig. 6:**
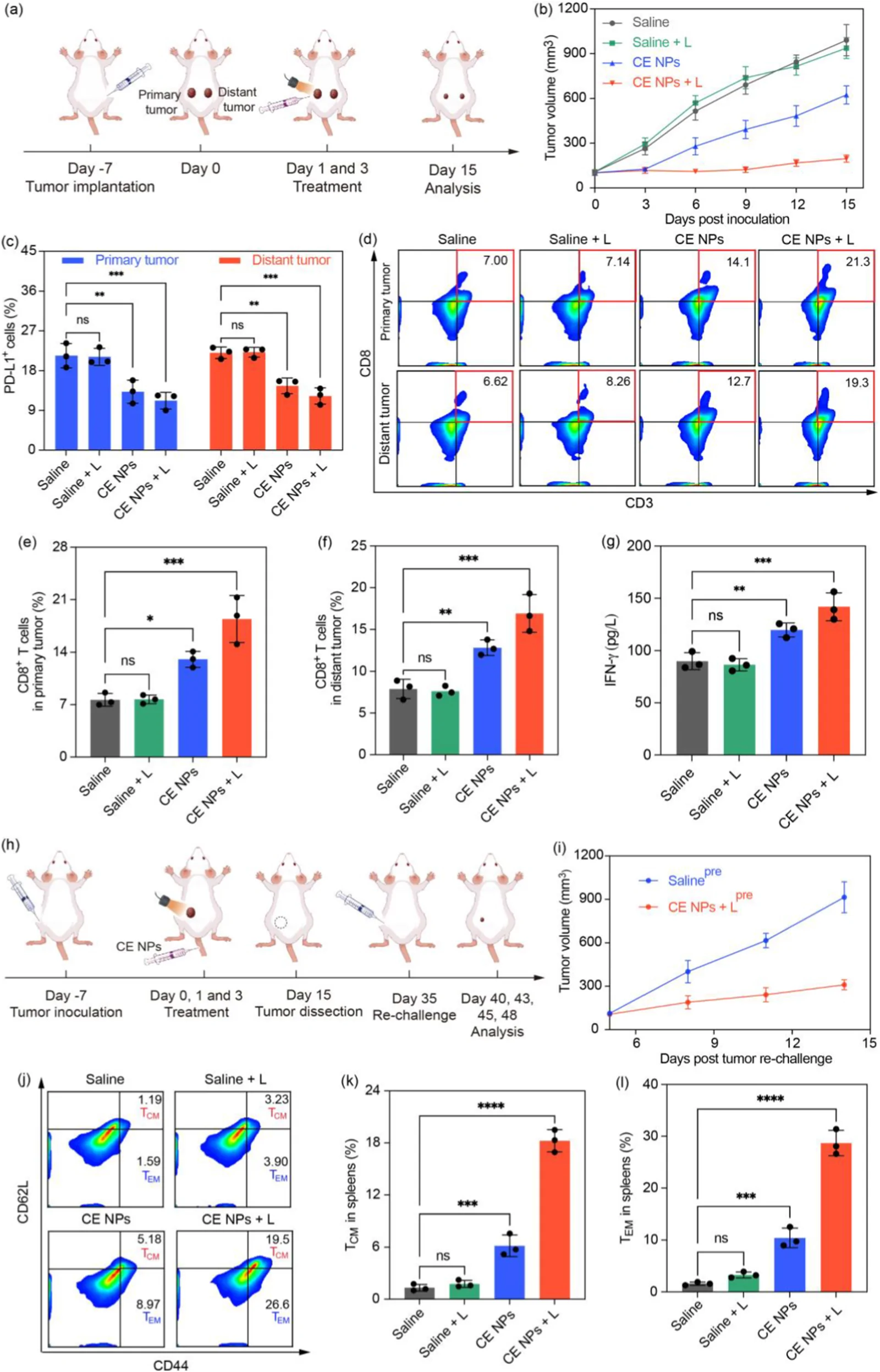
Systemic antitumor immunity, abscopal effect, and immune memory induced by CE NPs-mediated photoimmunotherapy. (a) Schematic illustration of the bilateral LLC tumor model and treatment regimen. Primary tumors and distant tumors were established on opposite flanks. Mice received the indicated treatments (Saline, Saline + L, CE NPs, and CE NPs + L) as indicated. (b) Growth curves of distant tumors in the bilateral tumor-bearing mice after different treatments. (c) Flow cytometry quantification of PD-L1^+^ cells in primary tumors (left) and distant tumors (right) after different treatments. (d) Representative flow cytometry plots of tumor-infiltrating CD8^+^ T cells (gated on CD3^+^ T cells) in primary tumors (upper) and distant tumors (lower) across different groups. Quantification of CD8^+^ T cells in primary tumors (e) and distant tumors (f) corresponding to (d). (g) IFN-γ levels measured after different treatments. (h) Schematic illustration of the tumor rechallenge model to evaluate long-term immune memory. Mice in both the Saline and CE NPs + light groups underwent surgical resection of the primary tumor after treatment, followed by a 20-day recovery period and subsequent tumor rechallenge. (i) Tumor growth curves after rechallenge in mice previously receiving Saline or CE NPs + L treatment. (j) Representative flow cytometry plots of central memory T cells (T_CM_, CD44^+^CD62L^+^) and effector memory T cells (T_EM_, CD44^+^CD62L^−^) in spleens across different groups. Quantification of splenic T_CM_ cells (k) and splenic T_EM_ cells (l) corresponding to (j). Light parameters: 660 nm, 0.5 W/cm^2^, 5 min, 150 J/cm^2^. Quantitative data are presented as mean ± SD. Distant-tumor growth curves in (b) and tumor-rechallenge growth curves in (i) were analyzed using two-way repeated-measures ANOVA. For single-time-point comparisons among multiple treatment groups in (c), (e-g), (k), and (l), one-way ANOVA was used. Statistical significance was analyzed as indicated, n = 3; ns, not significant; *P < 0.05, **P < 0.01, ***P < 0.001, ****P < 0.0001.

To determine whether CE NPs could elicit systemic antitumor immunity beyond local tumor ablation, we established a bilateral LLC tumor model consisting of a primary tumor and a contralateral distant tumor, followed with different treatments as illustrated in Fig. 6a. Tumor growth at the distant site serves as a readout of systemic antitumor immunity. Compared to Saline group or Saline + L group, CE NPs showed moderate tumor growth inhibition while CE NPs + L inhibited tumor growth most effectively among all the groups (Fig. 6b), indicating that CE NPs + L produced a robust systemic therapeutic outcome consistent with an abscopal-like effect [40].

Given that immune escape via PD-L1 is a key barrier to systemic tumor inhibition, we next quantified PD-L1^+^ cells in both tumor sites under different treatments. Flow cytometry analysis revealed that CE NPs treatment significantly reduced the fraction of PD-L1^+^ cells in the primary tumor and also in distant tumor with or without light illumination (Fig. 6c), mainly attributing to the fact that acetate mediated metabolism downregulates PD-L1 level in lung cancer [29]. These results confirm CE NPs not only modulates tumor-intrinsic immune evasion locally, but also reshapes the immunosuppression at distal lesions.

We next assessed whether these tumor-intrinsic changes induced by CE NPs have influence on immune surveillance. Flow cytometry results showed an increase in CD8^+^ T cells (CD3^+^CD8^+^) within both the primary (upper) and distant (lower) tumors after CE NPs treatment, and this effect was further amplified by 660-nm light activation (Fig. 6d).

Further quantification confirmed a significant elevation of CD8^+^ T cells in the primary tumor (Fig. 6e) and critically in the distant tumor (Fig. 6f) in CE NPs + L group.

These results demonstrate that CE NPs + L not only induced intratumoral immune response, but also initiated immune priming to restrain distal tumor growth. To substantiate T-cell activation in vivo, we measured IFN-γ, a key effector cytokine associated with cytotoxic T-cell responses. Indeed, CE NPs + L increased IFN-γ levels most while CE NPs alone enhanced IFN-γ levels partially compared to control groups (Fig. 6g), indicating the increased CD8^+^ T-cell infiltration induced by CE NPs + L treatment for anti-tumor immunotherapy.

Finally, to investigate whether CE NPs + L could induce durable antitumor immune memory, primary tumors were surgically resected after 15-day treatment period in both the Saline and CE NPs + light groups. Following a 20-day recovery period, mice were rechallenged with LLC cells and monitored for secondary tumor growth (Fig. 6h). Mice previously being pre-treated with CE NPs + L displayed marked tumor growth inhibition upon rechallenge compared with saline-pretreated controls (Fig. 6i), revealing the potential long-lasting protective immunity. Further flow cytometric results of splenic memory T cells revealed that CE NPs + L increased the ratio of both central memory T cells (T_CM_, CD44^+^CD62L^+^) and effector memory T cells (T_EM_, CD44^+^CD62L^−^) most, while CE NPs alone induced the partial expansion of both memory subsets (Fig. 6j–l). The enrichment of T_CM_ suggests the establishment of long-term immune persistence, whereas increased T_EM_ supports rapid recall potential, together providing a cellular basis for the observed resistance to tumor rechallenge [32]. Taken together, these results demonstrate that PDT-induced ICD and acetate metabolism induced by CE NPs + L reduces PD-L1-associated immune escape, enhances CD8^+^ T-cell infiltration, and induces robust T-cell memory, collectively conducting a new layer towards tumor growth inhibition and immune-priming for durable systemic antitumor immunity.

Beyond the current proof-of-concept study, further optimization of CE NPs will focus on improving their translational potential through comprehensive evaluation of pharmacokinetics, biodistribution, long-term biosafety, and scalable manufacturing. Such efforts will provide important guidance for advancing carrier-free photo-metabolic nanomedicines toward clinical applications.

## 3 Conclusion

In this study, we developed a carrier-free nanoregulator (CE NPs) by co-assembling Ce6 and EN4, a c-Myc inhibitor, integrating PDT and metabolic-immune reprogramming for NSCLC treatment. CE NPs generated singlet oxygen with light illumination to kill tumor cells and then induced ICD to initiate antitumor immune effect. Besides that, CE NPs directly inhibited c-Myc, which then decreased several key gene expressions for lung cancer proliferation and immune response, including MCT1, cyclin D1, LDHA, and PD-L1. First, decreasing MCT1 caused less acetate influx into LLC cells and further decreased c-Myc levels through c-Myc/MCT1/Acetyl-CoA closed-loop circuit. Second, downregulating cyclin D1 and LDHA prevented LLC cells from proliferation and glycolysis. More importantly, decreasing PD-L1 levels relieved immune evasion and boosted immunotherapy efficiency. Both in vitro and in vivo results confirmed CE NPs can effectively inhibited lung cancer growth. Overall, this work establishes a rational closed-loop blockade strategy that integrates c-Myc-centered acetate-metabolic regulation with PDT-induced ICD to suppress tumor growth and immune escape. Rather than simply combining two therapeutic modalities, this strategy couples photoactivated immune priming with disruption of a self-reinforcing metabolic/immune-evasion circuit, providing a mechanistic framework for the development of carrier-free photo-immunotherapeutic nanomedicines.

## CRediT authorship contribution statement

**Jianwei Zhu:** Writing – original draft, Investigation, Conceptualization. **Danming Dong:** Writing – original draft, Investigation, Data curation. **Jiayue Xu:** Resources, Investigation. **Xiaopeng Tang:** Writing – original draft, Funding acquisition. **Yuan Gao:** Investigation. **Jinbin Zhu:** Investigation. **Yini Zhuang:** Investigation. **Zhuang Lv:** Writing – review & editing, Supervision, Funding acquisition, Conceptualization. **Xiaoyuan Chen:** Writing – review & editing, Supervision, Investigation, Conceptualization. **Jianhua Zou:** Writing – review & editing, Methodology, Funding acquisition, Conceptualization. **Huaxia Shi:** Writing – review & editing, Supervision, Investigation, Funding acquisition.

## Declaration of competing interest

All authors declare no competing financial interest.

## Data Availability

Data will be made available on request.
