# Supplementary material for: Carrier-free nanomodulators reprogramming acetate metabolism for cancer photo-immunotherapy via c-Myc inhibition-induced closed-loop regulatory circuit
Source: Mater Today Bio. 2026 Sep 10;40:103660. doi: 10.1016/j.mtbio.2026.103660 (PMC13594637; doi:10.1016/j.mtbio.2026.103660)
Supplement: Supplementary file 1 [file mmc1.docx]

**Carrier-free Nanomodulators Reprogramming Acetate Acid Metabolism for Cancer Photo-immunotherapy via c-Myc inhibition-induced Close-cycle Blockade**

*Jianwei Zhu^#a*^, Danming Dong^#a^, Jiayue Xu ^a^, Xiaopeng Tang ^a^, Yuan Gao ^a^, Jinbin Zhu ^a^, Yini Zhuang ^a^, Pei Zhang^b^, Zhuang Lv*^b^, Xiaoyuan Chen*^c^, Jianhua Zou*^d^, Huaxia Shi** *^a^*

a. Department of Biochemistry, School of Life Sciences, Nanjing Normal University, Nanjing, Jiangsu 210046, China.

Email: jianweizhu@njnu.edu.cn; huaxia2022@nnu.edu.cn

b. Jiangsu Key Laboratory of New Drug Research and Clinical Pharmacy, Xuzhou Medical University, Xuzhou, Jiangsu 221004, China.

Email: 100002020021@xzhmu.edu.cn

c. Center for Nuclear Medicine and Molecular Imaging (CNMMI), Shandong Cancer Hospital and Institute, Jinan, China.

Email: [chen9647@gmail.com](mailto:chen9647@gmail.com).

d. Department of Biochemistry, School of Medicine, Southeast University, Nanjing, 210009, Jiangsu, China. Email: [zoujh93@seu.edu.cn](mailto:zoujh93@seu.edu.cn)

*^#^ The authors contribute equally.*

**Materials and reagents**

Chlorin e6 (Ce6) was purchased from Macklin Inc. TRIzol™ Reagent was purchased from Invitrogen™. 9, 10-anthracenedipropanoic acid (ABDA) were purchased from Bide Pharmatech Ltd. (China). DMEM medium and fetal bovine serum (FBS) were purchased from SenBeiJia Biological Technology Co., Ltd., China. Penicillin/streptomycin (P/S) and 0.25% trypsin were purchased from Wuhan ServiceBio Technology Co., Ltd. (China). Annexin V-FITC/PI Cell Apoptosis Kit, ROS Assay kit, and CCK8 Assay Kit were purchased from Beyotime Biotechnology (Shanghai, China). 2-(4- amidinophenyl)-1H-indole-6-carboxamidine (DAPI) was purchased from Solarbio, Co., Ltd. (Beijing, China). The primary antibodies used β-actin, GAPDH, PD-L1, LDHA,and Cyclin D1 antibody were purchased from Abclonal Technology (Wuhan, China). The primary antibodies used Bax, Bcl-2, Caspase3, Cytochrome c, PARP-1, c-Myc, MCT1 antibody were purchased from Proteintech (Wuhan, China). PE anti-mouse CD274, FITC anti-mouse CD11c, PE anti-mouse CD80, APC anti-mouse CD86 antibody were purchased from BioLegend. One-Step PAGE Gel Fast Preparation Kit and FlysisAmp Cells-to-CT 1-Step SYBR Green Kit were purchased from Vazyme (China).

**Preparation and charaterization of CE NPs**

Carrier-free CE nanoparticles (CE NPs) were prepared according to previously reported methods. Typically, Ce6 and EN4 were separately dissolved in DMSO to obtain stock solutions. Ce6 and EN4 were then mixed at different molar ratios (1:1, 1:2, or 1:3) to form a homogeneous organic mixture. The as-obtained mixture was added dropwise into deionized water under sonication. The mixture was continued to be sonicated for another 30 min. Then, the mixture was dialyzed against deionized water for one day, followed by being passed through a 0.22 µm syringe and stored at 4 °C for subsequent experiments.

The morphology of CE NPs was observed by transmission electron microscopy (TEM). The hydrodynamic size distribution and polydispersity index (PDI) were measured by dynamic light scattering (DLS), and the zeta potential was determined using a zeta potential analyzer. Unless otherwise stated, samples were appropriately diluted with deionized water or PBS prior to measurement, and data were collected at room temperature.

**UV-vis and fluorescence spectrum**

The UV-vis absorbance spectra of CE NPs in PBS or DMSO was recorded using a UV-vis spectrophotometer. Fluorescence emission spectra of CE NPs in PBS or DMSO was measured on a fluorescence spectrometer. To study the intermolecular interactions for CE NPs formation, the nanoparticles were incubated with NaCl, urea, or SDS at the indicated concentrations. Then, UV-vis absorbance spectra was recorded.

**^1^O_2_ production assay**

The photoinduced singlet oxygen generation of CE NPs was evaluated using 9,10-anthracenediyl-bis(methylene) dimalonic acid (ADBA) as a probe. Briefly, ABDA was mixed with CE NPs or PBS at the indicated concentrations and then exposed to NIR light illumination for different time (0 s, 30 s, 60 s, 90 s, 120 s, and 150 s). Then, fluorescence of ADBA was measured on a fluorescence spectrometer.

**Cell lines and animals**

LLC cells were cultured in DMEM with 10% FBS and 1% P/S at 37°C with 5% CO_2_. When cell density reached 80%-90%, cells were digested with trypsin, and then sub-cultured or inoculated in cell plates for subsequent experiments. C57BL/6 mice (4-6 weeks, male) were pursued from GemPharmatech (Nanjing, China) and raised in SPF animal rooms. All animal experiments reported were performed under the guidelines of the Committee of Animal Experimentation and the Ethics Committee, Nanjing Normal University.

**Cellular internalization of CE NPs in vitro**

For CLSM imaging, a cover slide was placed in the bottom of each well of a 24-well plate. LLC cells (1x10^5^) were inoculated into each well and incubated at 37°C overnight. Then, cells in each well were treated with CE NPs for 0h, 2h, 6h, and 12h, respectively. Next, the culture medium was removed and wash with PBS for three time followed by incubation with fresh PBS. The cover slide was placed on the microslide and the cell nuclei were stained with DAPI. Subsequently, images were collected by CLSM.

**Cell viability assay**

The cytotoxicity of CE NPs under different conditions was measured by CCK8 assay. LLC cells were inoculated into 96-well plates with a density of 3x10^3^ cells/well and cultured overnight. Cells were then treated with CE NPs at indicated concentrations with or without NIR light illumination. After that, cells in each group continued to be cultured for another 24 h, followed by the addition of 10 µL of CCK8 solution and being incubated for another 1 h. Subsequently, the absorbance at 450 nm was read with a microplate reader.

**Colony formation assay**

LLC cells were seeded in 6-well plates (5 × 10⁵ cells/well) and cultured to ~70% confluence. Cells were then treated with CE NPs (5 μM) or PBS for 12 h, followed by NIR irradiation for 5 min (light group) or no irradiation (dark group), and further incubated for another 12 h. After treatment, cells were harvested, dissociated into single-cell suspensions, and re-seeded into fresh 6-well plates at 1 × 10³ cells/well. Cells were cultured for 14 days with medium changed every 2-3 days. Colonies were fixed with 4% paraformaldehyde (37 °C, 15 min) and stained with crystal violet (20 min, room temperature). Plates were imaged after drying, and colonies were counted for quantification.

**Detection of intracellular ROS**

LLC cells were inoculated into 6-well plates with a density of 5x10^5^ cells/well and cultured overnight. Then, CE NPs were added in and incubated for 12h, respectively. For light treatment, each group was lighted for 2 min. All groups were stained with DCFH-DA for half an hour, and ROS production was recorded by inverted fluorescence microscopy.

**Apoptosis analysis by flow cytometry**

Cellular apoptosis was assessed with an Annexin V-FITC apoptosis detection kit. In brief, LLC cells were seeded into 12-well plate at 3x10^5^ cells per cell and incubated at 37 °C overnight. After that, cells in each well were treated with CE NPs or PBS with or without light illumination, followed by being incubating for another 12h. Next, cells in each well were washed with PBS three times and incubated with Annexin/ PI reagent in the dark for 15 min at 25°C. Finally, cells in each well were immediately measured by FCM.

**Western blotting analysis**

After LLC cells were treated with CE NPs or PBS with or without light illimunation, total cell proteins were extracted by RIPA lysis buffer supplemented with protease and phosphatase inhibitors. Concentration of protein lysates were determined by the Bradford method and denatured at 100°C. Protein samples were separated by sodium dodecyl sulfate-polyacrylamide gel electrophoresis (SDS-PAGE) and then transferred to 0.45 µM PVDF membranes (Merck Millipore) at 4°C. After being blocked with 5% skimmed milk, the membranes were incubated with diluted primary antibodies at 4 °C overnight and then incubated with horseradish peroxidase (HRP)-conjugated secondary antibodies at room temperature for an hour. The bands were detected using an ECL detection reagent (ServiceBio, China) by Chemiluminescence imager.

**Immunofluorescence staining of CRT and HMGB1.**

LLC cells were seeded in confocal dishes (3 × 10³ cells/dish) and cultured to ~70% confluence. Cells were then treated with CE NPs or PBS for 12 h, followed by NIR light illumination for 5 min, and further incubated for another 12 h. After treatment, cells were fixed with 4% paraformaldehyde (37 °C, 20 min) and washed with PBS. For HMGB1 staining, cells were permeabilized with 0.25% Triton X-100 (10 min, room temperature), whereas permeabilization was omitted for CRT staining. After blocking with 5% FBS in PBS (1 h), cells were incubated with primary antibodies against CRT or HMGB1 (1:250, 4 °C overnight), followed by fluorophore-conjugated secondary antibodies (1 h, room temperature, protected from light). Nuclei were counterstained with Hoechst (2 μg/mL, 10 min), and images were acquired using a confocal laser scanning microscope.

**BMDC generation and in vitro maturation assay.** Bone marrow cells were harvested from femurs and tibias of 8-week-old C57BL/6 mice by flushing with complete medium, followed by filtration, red blood cell lysis, and centrifugation. To generate immature BMDCs, bone marrow cells were cultured in complete medium supplemented with GM-CSF, IL-4, and β-mercaptoethanol, with medium refreshed as needed; immature BMDCs were collected on day 8 for subsequent experiments. For the BMDC maturation assay, LLC cells were seeded in 6-well plates (5 × 10⁵ cells/well) and treated with CE NPs (or PBS) with/without NIR irradiation as described above. Treated LLC cells (5 × 10⁵ cells) were transferred to the upper chamber of Transwell inserts, while immature BMDCs (1 × 10⁶ cells) were seeded in the lower chamber and co-cultured for 24 h. BMDCs were then collected from the lower chamber, washed with cold PBS, and stained with fluorophore-conjugated antibodies against CD11c, CD80, and CD86 (4 °C, 60 min, protected from light). BMDC maturation was analyzed by flow cytometry as the percentage of CD11c⁺CD80⁺CD86⁺ cells.

Top of Form

**LC-MS quantification of acetate, acetyl-CoA, and lactate in vitro**

LLC cells were treated under different conditions, washed with ice-cold PBS, and rapidly quenched on ice. Cellular metabolites were extracted using pre-chilled organic solvent (e.g., 80% methanol or methanol/acetonitrile/water), followed by vortexing and incubation at -20 °C to maximize extraction. Lysates were centrifuged with high speed at 4 °C to remove debris, and the supernatants were collected, dried under vacuum, and reconstituted in LC/MS-grade solvent prior to analysis. Targeted LC-MS/MS was performed in multiple reaction monitoring (MRM) mode using authentic standards to quantify acetate, acetyl-CoA, and lactate.

**IL-2 and IFN-γ level quantification *in vitro***

LLC-OVA cells were pre-treated with acetate under low-glucose conditions as indicated, followed by incubation with CE NPs (or PBS) with/without NIR light illumination. After treatment, LLC-OVA cells were washed to remove residual reagents and then co-cultured with OT-1 mouse-derived CD8⁺ T cells for the indicated time. For gene-expression analysis, CD8⁺ T cells were collected, and total RNA was extracted and reverse-transcribed into cDNA. Il2 and Ifng mRNA levels were quantified by RT-qPCR and normalized to a housekeeping gene (Gapdh) using the 2^-ΔΔCt^ method. For cytokine secretion analysis, culture supernatants from the co-culture system were harvested, and IL-2 and IFN-γ concentrations were measured using commercial ELISA kits according to the manufacturers’ instructions.

**RNA sequencing**

After LLC cells treated with CE NPs with/ without light illumination, total RNA in each group was extracted by using TRIzol. Total RNA concentration was measured using a Nanodrop. The RNA-seq library was conducted following the manufacturer’s protocol (Vazyme, China). Briefly, the first cDNA fragment was generated by the reverse random primer, and the second cDNA was synthesized by adding dUTP. The double-stranded cDNA ends were repaired with A tails and connected to Illumina matching adaptors, and the final library was obtained by PCR amplification. An Agilent 2100 bioanalyzer was applied for quality control, qPCR was used for library quantification, and an Illumina HiSeq 4000 instrument was used for sequencing. All RNA-seq services were performed by ServiceBio (Wuhan, China).

**Evaluation of therapeutic efficacy *in vivo***

First, a subcutaneous tumor model was conducted. Around 1x10^7^ LLC cells were subcutaneously injected into C57BL/6 mice (4-6 weeks). As the tumor size reached out 100 mm^3^, the mice were randomly divided into four groups: Saline, Saline + NIR, CE NPs, and CE NPs + NIR. Then, 100 µL of drugs were injected into the mice intravenously, followed by light. Mice body weight and tumor size were recorded every two days. Tumor size was calculated as below:

Volume = Length × Width^2^/ 2

On day 12, all mice in each group were euthanized, tumors and main organs (liver, heart, spleen, kidney, and lung) were collected for further analysis.

**Histopathological analysis and Immunohistochemistry (IHC) analysis**

The solid tumor was harvested from tumor-bearing mice on the 12^th^ day of tumor inoculation for histological observation by standard H&E staining. For H&E staining, the excised tumor and organs were fixed in 4% paraformaldehyde solution, embedded in paraffin, sectioned, and stained with H&E. The sections were then observed under a fluorescence microscope.

IHC staining sections were placed in citrate sodium buffer overnight at 60°C after dewaxing for antigen recovery. Afterwards, sections were immersed in 3% H_2_O_2_ for 10 min and blocked with 5% bovine serum albumin (BSA) at room temperature for 1 h. The sections were incubated with corresponding primary antibodies overnight at 4°C, and then incubated with biotin labeled secondary antibody at room temperature for 1 h. DAB reagent was applied eventually. IHC images were obtained via the microscope.

**Immune repsonse analysis post-treatment in vivo**

Subcutaneous LLC tumor-bearing mice were established and treated as indicated above. For analysis of systemic antigen-presenting responses, mice were euthanized on day 12 post-treatment and tumor-draining lymph nodes (TDLNs) and spleens were collected. Tissues were enzymatically dissociated using collagenase and DNase I, filtered through a cell strainer to obtain single-cell suspensions, and spleen samples were subjected to red blood cell lysis followed by PBS washes. Cells were stained with fluorophore-conjugated antibodies against CD11c, CD80, and CD86 (4 °C, 60 min, protected from light) and analyzed by flow cytometry; mature DCs were quantified as CD11c⁺CD80⁺CD86⁺ cells.

For T-cell profiling, mice were euthanized on day 12 post-treatment and tumors and spleens were harvested and processed into single-cell suspensions using collagenase/DNase digestion and filtration as above (with red blood cell lysis for spleen samples). Cells were stained with antibodies against CD3, CD4, and CD8a (4 °C, 60 min, protected from light), washed, resuspended in PBS, and analyzed by flow cytometry to quantify CD3⁺ T cells, CD3⁺CD4⁺ T cells, and CD3⁺CD8⁺ T cells.

Top of Form

**Therapeutic analysis of distal tumor in bilateral tumor model**

A bilateral subcutaneous LLC tumor model was established by inoculating LLC cells into the right flank (primary tumor) and the contralateral left flank (distant tumor). When the primary tumors reached approximately 100 mm³, mice were randomized into four groups: Saline, Saline + light, CE NPs, and CE NPs + light. Saline or CE NPs were administered intratumorally into the primary tumor only. Where indicated, only the primary tumor was exposed to 660-nm light irradiation. The contralateral distant tumor received neither intratumoral injection nor light irradiation and was monitored throughout the experiment to evaluate the distant antitumor response. Mice in each group reacieve treatment as indicated for 15 days. Tumor length and width in distant tumor were recorded at indicated time points. Tumor size was calculated as below:

Volume = length × Width^2^/ 2

On day 15, all mice in each group were euthanized, both tumors were collected for further analysis.

**PD-L1^+^ cells and CD8^+^ T cells quantification in Bilateral tumor model**

Primary and distant tumors were harvested at the endpoint, minced, and enzymatically digested (collagenase + DNase I) to generate single-cell suspensions, followed by filtration through a cell strainer. Cells were washed and stained with fluorophore-conjugated antibodies against PD-L1. The percentage of PD-L1⁺ cells was analyzed by flow cytometry and further quantified. Cell suspensions from primary and distant tumors were prepared as described above. Cells were stained with antibodies against CD3 and CD8. Tumor-infiltrating CD8⁺ T cells were gated as CD3⁺CD8⁺ cells.

**Immune memory evaluation in vivo**

C57BL/6 mice were subcutaneously inoculated with LLC cells into the right flank to establish the primary tumor. When tumor volume reached to ~ 100 mm^3^, mice in each group was treated with Saline or CE NPs + NIR. After a 15-day treatment, mice in both the Saline and CE NPs + light groups underwent the same surgical resection of the primary tumor. Mice were then allowed to recover for 20 days before tumor rechallenge. Then, mice were re-inoculated with LLC cells to initiate tumor re-challenge, followed by tumor-volume monitoring and immune response analysis at the indicated time points.

After tumor re-challenge, tumor sizes (length and width) were measured at the indicated days. Tumor size was calculated as below:

Volume = length x Width^2^/ 2

On the endpoint, all mice in each group were euthanized, spleens were harvested and processed into single-cell suspensions, followed by red blood cell lysis. Cells were then stained with fluorophore-conjugated antibodies against CD44 and CD62L (4 °C, 60 min, protected from light), washed, and analyzed by flow cytometry.

**Statistical analysis**

Quantitative data are presented as mean ± standard deviation (SD). Each experiments were repeated at least 3 times independently. The exact biological sample size (n) is specified in the corresponding figure legends. For individual comparisons, one-way ANOVA and a two-tailed, unpaired Student’s t-test were performed using GraphPad Prism software.

Figure S1. Representative DLS size distribution of self-assembled nanoparticles between Ce6 and EN4 prepared at different feed ratios: top, 1:2; bottom, 1:3.

Figure S2. Stability of CE NPs (Ce6: EN4 = 1: 1) over 7 days in H_2_O or PBS. Hydrodynamic size (top) and polydispersity index (PDI) were measured by dynamic light scattering (n = 3).

Figure S3. Flow cytometry analysis of intracellular uptake of CE NPs in LLC cells at different time plots (0h, 2h, 6h, and 12h).

Figure S4. Quantification of intracellular DCF fluorescence intensity (a.u.) corresponding to the representative images in Figure 2d. Fluorescence intensity was quantified using ImageJ from three independent experiments (n = 3). Data are presented as mean ± SD. Statistical significance was analyzed by one-way ANOVA. ns, not significant; **P < 0.0001.

Figure S5. Quantification of apoptosis percentage of LLC cells with different treatment from Figure 2e.

Figure S6. Quantificative analysis of western blotting results for Bax, Bcl-2, Caspase-3, Cyt-c, and PARP-1 in LLC cells under different treatments. Protein levels were normalized to β-actin and expressed relative to the control group. Data are presented as mean ± SD from three independent experiments (n = 3).

Figure S7. Quantificative analysis of western blotting results for c-Myc, MCT1, PD-L1, Cyclin D1, and LDHA in LLC cells under different treatments. Protein levels were normalized to β-actin and expressed relative to the control group. Data are presented as mean ± SD from three independent experiments (n = 3).

Figure S8. Quantification of fluorescence intensity of LLC tumor bearing mice at different times post-injection of CE NPs intravenously.

Figure S9. Serum AST, ALT, BUN, and CRE levels in mice from different treatment groups. Data are expressed as mean ± SD. ns, no significant difference.

Figure S10. Representative H&E-stained tissue sections of heart, liver, spleen, lung, and kidney collected from mice treated with Saline, Saline + L, CE NPs, or CE NPs + L.

Figure S11. Quantification of acetyl-CoA level in harvested LLC tumor tissues after different treatments. Mice were treated with Saline, Saline + light, CE NPs, or CE NPs + light as indicated, and tumors were collected after treatment for metabolite analysis. Data are presented as mean ± SD. Statistical significance was determined using one-way ANOVA. ns, not significant; *P < 0.05.

Figure S12. Quantification of acetate level in harvested LLC tumor tissues after different treatments. Mice were treated with Saline, Saline + light, CE NPs, or CE NPs + light as indicated, and tumors were collected after treatment for metabolite analysis. Data are presented as mean ± SD. Statistical significance was determined using one-way ANOVA. ns, not significant; *P < 0.05.

Figure S13. Representative immunohistochemical (IHC) images of HMGB1 (top row) and calreticulin (CRT) (bottom row) staining in tumor sections harvested from mice treated with Saline, Saline + L, CE NPs, and CE NPs + L.

Figure S14. Serum IFN-γ (a) and serum TNF-α (b) levels in tumor-bearing mice treated with Saline, Saline + L, CE NPs, or CE NPs + L, quantified by ELISA. Data are presented as mean ± SD. Statistical significance was analyzed as indicated: ns, not significant; *P < 0.05, **P < 0.01, ***P < 0.001.
